# Methylation modifications in tRNA and associated disorders: Current research and potential therapeutic targets

**DOI:** 10.1111/cpr.13692

**Published:** 2024-06-28

**Authors:** Zhijing Wu, Ruixin Zhou, Baizao Li, Mingyu Cao, Wenlong Wang, Xinying Li

**Affiliations:** ^1^ Department of General Surgery, Xiangya Hospital Central South University Changsha Hunan China; ^2^ National Clinical Research Center for Geriatric Disorders, Xiangya Hospital Central South University Changsha Hunan China; ^3^ Department of Breast Surgery, Xiangya Hospital Central South University Changsha Hunan China; ^4^ Clinical Research Center for Breast Cancer in Hunan Province Changsha Hunan China

## Abstract

High‐throughput sequencing has sparked increased research interest in RNA modifications, particularly tRNA methylation, and its connection to various diseases. However, the precise mechanisms underpinning the development of these diseases remain largely elusive. This review sheds light on the roles of several tRNA methylations (m1A, m3C, m5C, m1G, m2G, m7G, m5U, and Nm) in diverse biological functions, including metabolic processing, stability, protein interactions, and mitochondrial activities. It further outlines diseases linked to aberrant tRNA modifications, related enzymes, and potential underlying mechanisms. Moreover, disruptions in tRNA regulation and abnormalities in tRNA‐derived small RNAs (tsRNAs) contribute to disease pathogenesis, highlighting their potential as biomarkers for disease diagnosis. The review also delves into the exploration of drugs development targeting tRNA methylation enzymes, emphasizing the therapeutic prospects of modulating these processes. Continued research is imperative for a comprehensive comprehension and integration of these molecular mechanisms in disease diagnosis and treatment.

## Graphical abstract

Dysregulation of tRNA methylation modifications and their associated enzymes has been implicated in a spectrum of diseases, including cancer and neurological disorders. Further exploration is warranted to fully elucidate the diagnostic and therapeutic potential of tRNA methylations in human diseases.

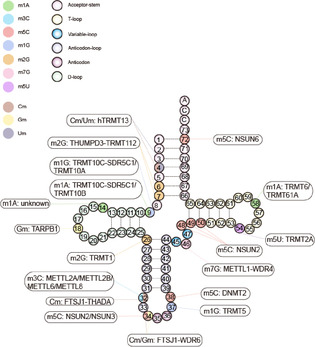

## 1 INTRODUCTION

Chemical modifications are extensively utilized within human cells to efficiently regulate the function and structure of biological macromolecules including DNA, RNA, and proteins. These modifications exhibit a diverse range within proteins and RNA.^1^ Any aberrations in these modifications within molecules can lead to pathological consequences. For instance, DNA hypomethylation can elevate the mutation rate and is strongly associated with the occurrence of cancer.^2^, ^3^ Also, hypomodification of tRNAs can trigger the generation of tRNA‐derived small RNAs (tsRNAs), which have been associated with cancer, neurodegenerative disorders, and metabolic disorders.^4^ Furthermore, various types of post‐translational modifications of proteins exist, capable of altering the structure, function, and interactions of proteins. Anomalies in protein O‐glucosyltransferase modifications, for example, can contribute to diseases such as leukaemia, pancreatic dysfunction, and Alzheimer's disease.^5^ Current research primarily concentrates on the dysregulation of protein and DNA modifications within the realms of pathology and pharmacology. Substantial advancements have been achieved in cancer‐targeted therapy, with numerous ongoing clinical trials. However, RNA modifications have received relatively little attention due to the constraints of detection technology. With the recent emergence of novel sequencing technologies facilitated by high‐throughput sequencing (HTS), RNA modification has garnered significant research interest.

**FIGURE 1:**
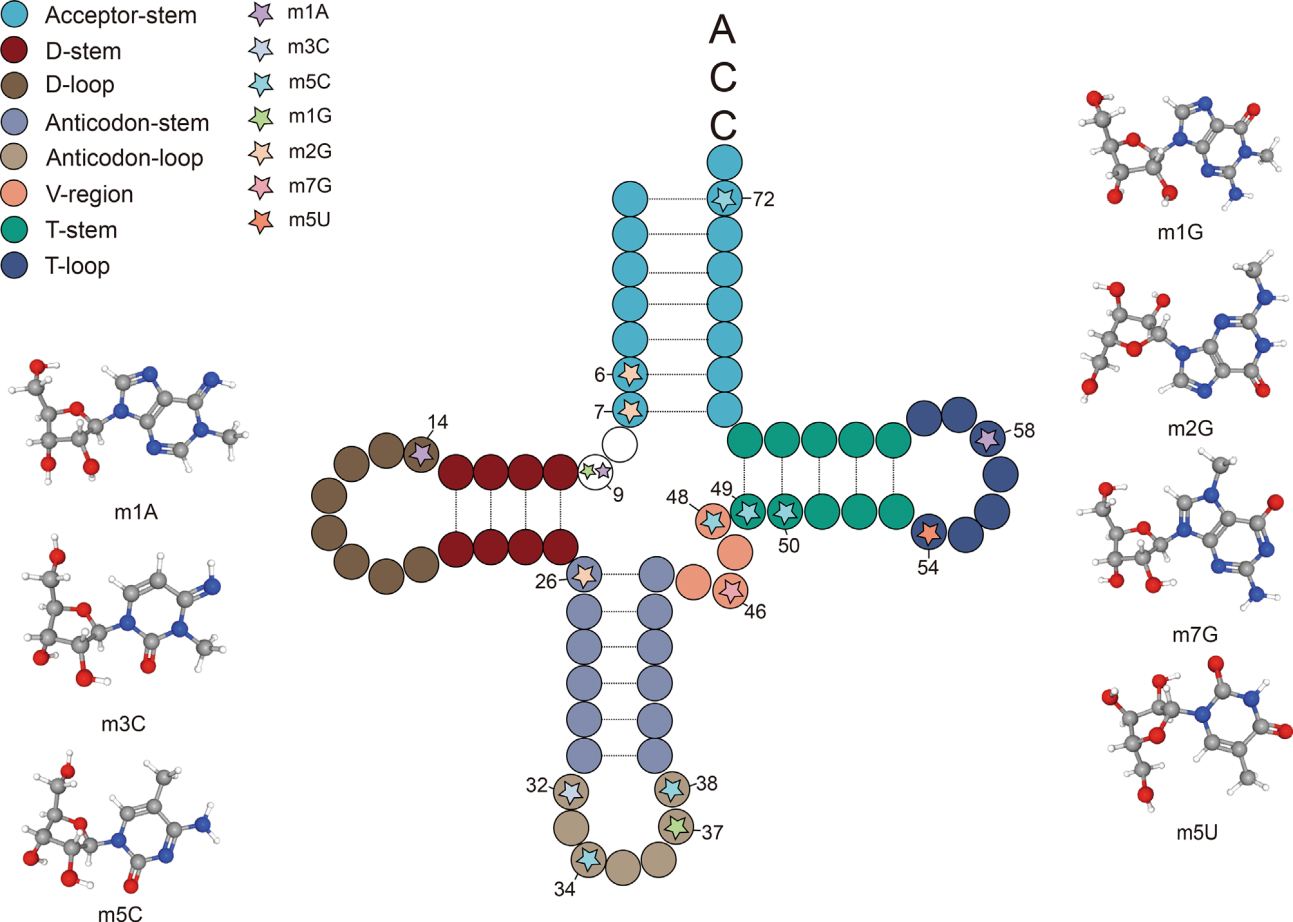
The molecular structure and methylation modifications of transfer RNA (tRNA) encompass various common methylation sites, including modifications in bases such as N1‐methyladenosine (m1A), N3‐methylcytosine (m3C), N5‐methylcytosine (m5C), N1‐methylguanosine (m1G), N2‐methylguanosine (m2G) and N7‐Methylguanosine (m7G) and 2′‐O‐methylation (2′‐O‐methylcytidine (Cm), 2′‐Omethylguanosine (Gm), 2′‐O‐methyluridine (Um)). These specific methylation sites in tRNA are characterized by distinct sequences, and the detailed chemical composition of the methylation groups is depicted.

tRNA is a ubiquitous molecule in human cells acting as an adapter molecule in protein biosynthesis. Its role is crucial for maintaining normal cellular activities. Among RNA molecules, tRNA undergoes the most modifications, with the anticodon loop and tRNA core region being the two focal points of these modifications.^6^ The anticodon loop plays a crucial role in regulating both translation accuracy and efficiency, while the body region of the tRNA influences its stability and overall structure.^7^ Methylation stands out as the most prevalent tRNA modification,^8^ a process catalysed by specific enzymes known as “Writers”—the tRNA methyltransferases. These enzymes utilize S adenosylmethionine (SAM) as a methyl donor to introduce modifications such as, N1‐methyladenosine (m1A), N3‐methylcytosine (m3C), N5‐methylcytosine (m5C), N1‐methylguanosine (m1G), N2‐methylguanosine (m2G), N7‐methylguanosine (m7G), N5‐methyluridine (m5U) and 2‐O‐methylation (Nm). In addition to the “Writers”, there are enzymes known as “Erasers” that function to demethylate tRNA, as well as “Readers” that are responsible for recognizing the methylated sites and binding proteins (Figure 1).

The activity of tRNA methylases exhibits variability across different cellular states.^9^ Correspondingly, fluctuations in enzymatic activity lead to alterations in methylation levels. Consequently, the level of tRNA methylation is dynamically modulated in response to variations in cellular metabolite levels and environmental stresses.^10^ Dysregulation of tRNA can have detrimental effects, contributing to various human diseases, including neurological disorders and cancer (Table 1).^11^ Given its significant impact on pathological processes in numerous human diseases, tRNA methylation has emerged as promising target for prediction, treatment, and prognosis biomarkers in clinical settings.^4^, ^12^ In recent years, m5C and m7G have garnered attention. Therapeutic strategies involving the targeting of tRNA methylation sites or relevant enzymes have been developed, particularly in the context of cancer.^13^, ^14^

**TABLE 1:** The role of tRNA methylations in diseases.

Disease	Modifications	Enzyme	Regulation	Function	References
AD	m1A	TRMT10C, TRMT61A	Down‐regulation	Affect mitochondrial processing, leading to accumulation of precursor transcripts	36
Advanced NBL	m7G	METTL1/WDR4	Up‐regulation	METTL1 regulates tRNA m7G modification, tRNA expression, and overall mRNA translation	152
ARID and facial dysmorphia	m5C	NSUN2	Nonsense mutation	Induce changes in tissue‐specific protein expression, result in proteomic shifts at critical stages during brain development	221, 222
BLCA	m1A	TRMT6/61A	Up‐regulation	Associates with dysregulation in the tRFs targetome	27
	m1A, m3C, m1G	/	Up‐regulation	Functions as a biomarker for BLCA detection or follow‐up	100, 101
	m7G	METTL1/WDR4	Up‐regulation	Participates in the METTL1‐m7G‐EGFR/EFEMP1 axis in cancer development	153
BRCA	m1A and m3C	ALKBH3	Up‐regulation	Promotes cancer proliferation, migration and invasion via tDRs induction, enhances ribosome assembly and prevents apoptosis triggered by Cyt c	30
	m3C	METTL2A	Up‐regulation	Acts as an oncogene	52
Colorectal cancer	m3C	METTL8	Frameshift mutation	Causes premature stops of amino acid synthesis in the proteins	56
Contextual fear memory deficits	m5C	NSUN2	Down‐regulation	Leads to specific defects in the glycine codon and reduces the levels of glycine‐enriched proteins, alters glutamatergic neurotransmission to damage synaptic signalling at the pyramidal neurons in the prefrontal cortex	91
COVID‐19	m2,2G	TRMT1	Cleaved by SARS‐CoV‐2 main protease	Regulates protein translation or oxidative stress response and contributes to viral pathogenesis	131
DEEs	m3C	METTL6, METTL8	Down‐regulation	Inhibits stem cell self‐renewal, resulting in defective neuronal stem cells in human cerebral cortex	50, 53, 58
Diabetes	m1G	TRMT10A	Down‐regulation	Inhibits Trmt10A expression and reduces m1G, induces oxidative stress and triggers apoptosis in pancreatic β‐cells	106
DS	m5C	NSUN2	Down‐regulation	Upregulates tRFs, reduces translation rates, activates stress pathways, impair differentiation of NESCs, resulting in apoptosis and volume reduction of neuronal cells in the cortex, hippocampus, and striatum	88, 89, 90
ESCC	m7G	METTL1/WDR4	Up‐regulation	Promotes tumorigenesis via the RPTOR/ULK1/autophagy axis	146
GBM	m1A	TRMT6/61A	Up‐regulation	Regulates the translation of mRNAs encoding proteins involved in the tumorigenic process and increase the ability to form colonies or spheres in vitro	29
GC	m7G	/	Up‐regulation	Decreases immune cell infiltration, increases immune escape and dysfunction, positively correlates with survival time, clinical stage, and efficacy of immunotherapy	143
HCC	m1A	TRMT6/61A	Up‐regulation	Increases PPARδ translation, triggers cholesterol synthesis to activate Hedgehog signalling, drives self‐renewal of liver CSCs and tumorigenesis	28
	m3C	METTL6	Up‐regulation	Increases the level of cell adhesion proteins, promotes the proliferation, invasion and migration of HCC cells	54
	m7G	METTL1/WDR4	Up‐regulation	Participates in METTL1‐TGF‐β2‐PMN‐MDSC axis to form immunosuppressive environment and enhances the translation of SLUG/SNAIL to promote EMT, associates with advanced tumour stages and poor patient survival	141, 142
HNSCC	m5C	NSUN2	Up‐regulation	Interact with T cell activation status	84, 85
	m7G	METTL1/WDR4	Up‐regulation	Regulates global mRNA translation, including the PI3K/AKT/mTOR signalling pathway, and alter immune landscape	151
ICC	m7G	METTL1/WDR4	Up‐regulation	Selectively regulates the translation of oncogenic transcripts, including cell‐cycle and EGFR pathway genes, in m7G‐tRNA‐decoded codon‐frequency‐dependent mechanisms, associates with poor prognosis	147
ID	m2,2G	TRMT1	Missense variant	Impairs tRNA modification and reconstitution of enzymatic activity, leads to loss of function	130
LSCC	m3C	METTL8	Up‐regulation	Prevents cell cycle arrest and promotes the proliferation of cells	57
Lung cancer	m7G	METTL1/WDR4	Up‐regulation	Increases cell proliferation, colony formation, cell invasion, and tumorigenic capacities of lung cancer cells	150
MERRF	m1A	/	Missing in mt‐DNA	Maintains the translation elongation and stabilizes the selected nascent chains	35
NAFLD	m1G, m2G	/	Up‐regulation	Serves as a biomarker of NAFLD	104
NPC	m7G	METTL1/WDR4	Up‐regulation	Upregulates the WNT/β‐catenin signalling pathway with an upstream transcription factor ARNT to promote EMT and chemoresistance to cisplatin and docetaxel	149
NSXLID	Nm	FTSJ1	Nonsense mutation	Reduces the UUU codon and the steady‐state level of tRNA^Phe^ in the brain, activate the GAAC, exhibiting immature synaptic morphology and aberrant synaptic plasticity, associated with anxiety‐like and memory deficits	173, 174, 175
Oral cancer	m5C	NSUN3	Up‐regulation	Does not affect primary tumour growth but promotes efficient metastasis by protecting mitochondrial metabolism	86
Ovarian cancer	m1G	/	Up‐regulation	Contributes to the tumour microenvironment	103
Pancreatic cancer	m3C	METTL8	Up‐regulation	Enhance the respiratory chain activity and correlates with lower patient survival	48
Prostate cancer	m1A and m3C	ALKBH3	Up‐regulation	Promotes cancer proliferation, migration and invasion via induction of tDRs, facilitates the ribosome assembly and prevents apoptosis triggered by Cyt c	30
	m7G	METTL1	Up‐regulation	Inhibits small non‐coding RNAs derived from 5′‐tRNA fragments to suppress tumour growth, interferon pathway, and immune effectors	148

Abbreviations: AD, Alzheimer disease; ARID, autosomal‐recessive intellectual disability; ARNT, aryl hydrocarbon receptor nuclear translocator; BLCA, bladder cancer; BRCA, breast carcinoma; COVID‐19, coronavirus disease‐19; DEEs, Developmental and epileptic encephalopathies; DS, Dubowitz‐like syndrome; EMT, epithelial–mesenchymal transition; ESCC, oesophageal squamous cell carcinoma; GAAC, general amino acid control pathway; GBM, glioblastoma; GC, gastric cancer; HCC, hepatocellular carcinoma; HNSCC, head and neck squamous cell carcinoma; ICC, intrahepatic cholangiocarcinoma; ID, intellectual disability; LSCC, lung squamous cell carcinoma; MERRF, myoclonic epilepsy with ragged red fibres syndrome; NAFLD, nonalcoholic fatty liver disease; NBL, neuroblastoma; NESCs, neuroepithelial stem cells; NPC, nasopharyngeal carcinoma; NSXLID, non‐syndromic X‐linked intellectual disability; tDRs, tRNA‐derived small RNAs; tRFs, tRNA‐derived fragments.

Herein, we summarize the common types of tRNA methylations and describe their general features, associated enzymes, and biological functions. Furthermore, we outline the diseases linked to dysregulated methylation, elucidating the underlying mechanisms and pathways involved. Lastly, we assess the current limitations of tRNA methylations as diagnostic and therapeutic targets in human diseases.

## 2 tRNA METHYLATION

In tRNAs, a wide array of epigenetic methylation marks and their corresponding regulatory enzymes play pivotal roles in cellular functions and pathological processes. tRNA methylation includes m1A, m3C, m5C, m1G, m2G, m7G, m5U, and Nm. These modifications are introduced by a range of “writers”, including members of the tRNA Methyltransferase (TRMT) family, DNA methyltransferase (DNMT) family, Methyltransferase‐like (METTL) family and NOL1/NOP2/sun (NSUN) family, and removed by “erasers” like the AlkB Homologue (ALKBH) family and ten‐eleven translocator (TET) family. The role of “readers” is still not fully understood and necessitates further research for complete elucidation.

Dysregulation of these modifications is associated with a variety of pathological states, including cancer, neurological disorders, and metabolic abnormalities. This highlights the critical role of tRNA methylation in maintaining cellular homeostasis and underscores its potential as a target for therapeutic strategies.

### 2.1 N1‐methyladenosine (m1A) modification in tRNA

The m1A modification in tRNA, commonly located in the T‐loop and predominantly found at position 58, plays a role in modulating metabolic processing, stability, protein interactions, and mitochondrial activity.^15^ This modification is catalysed by “writers”, presented by TRMT6/TRMT61A. For instance, TRMT61B is responsible for introducing m1A58 in human mitochondrial tRNAs (mt‐tRNAs).^16^ Specifically, TRMT61B modifies m1A58 in mt‐tRNAs, while TRMT10A and TRMT10B independently methylate m1G9 and m1A9 in tRNA.^16^, ^17^, ^18^, ^19^ Meanwhile, the demethylation of m1A is carried out by the ALKBH family, which includes ALKBH1, ALKBH3, ALKBH7, and Fat Mass and Obesity‐Associated Protein (FTO).^20^, ^21^, ^22^ The ALKBH family comprises several homologues (ALKBH1‐8) involved in the oxidative demethylation of DNA and RNA.^23^ ALKBH1 is involved in a stress‐specific process that enhances tRNA unequal cleavage, and its depletion triggers the mitochondrial unfolded protein response (UPR).^21^ FTO, identified through genome‐wide association studies as related to fat mass and obesity, selectively demethylates N6‐methyladenosine (m6A) in mRNA and m1A in tRNA.^19^, ^24^ “Readers” like YTHDF1‐3 and YTHDC1 directly bind to m1A to facilitate these modifications.^25^ Additionally, ribozymes like MTR1 have recently also emerged, further expanding the scope of m1A modifications in tRNA machinery.^26^

**FIGURE 2:**
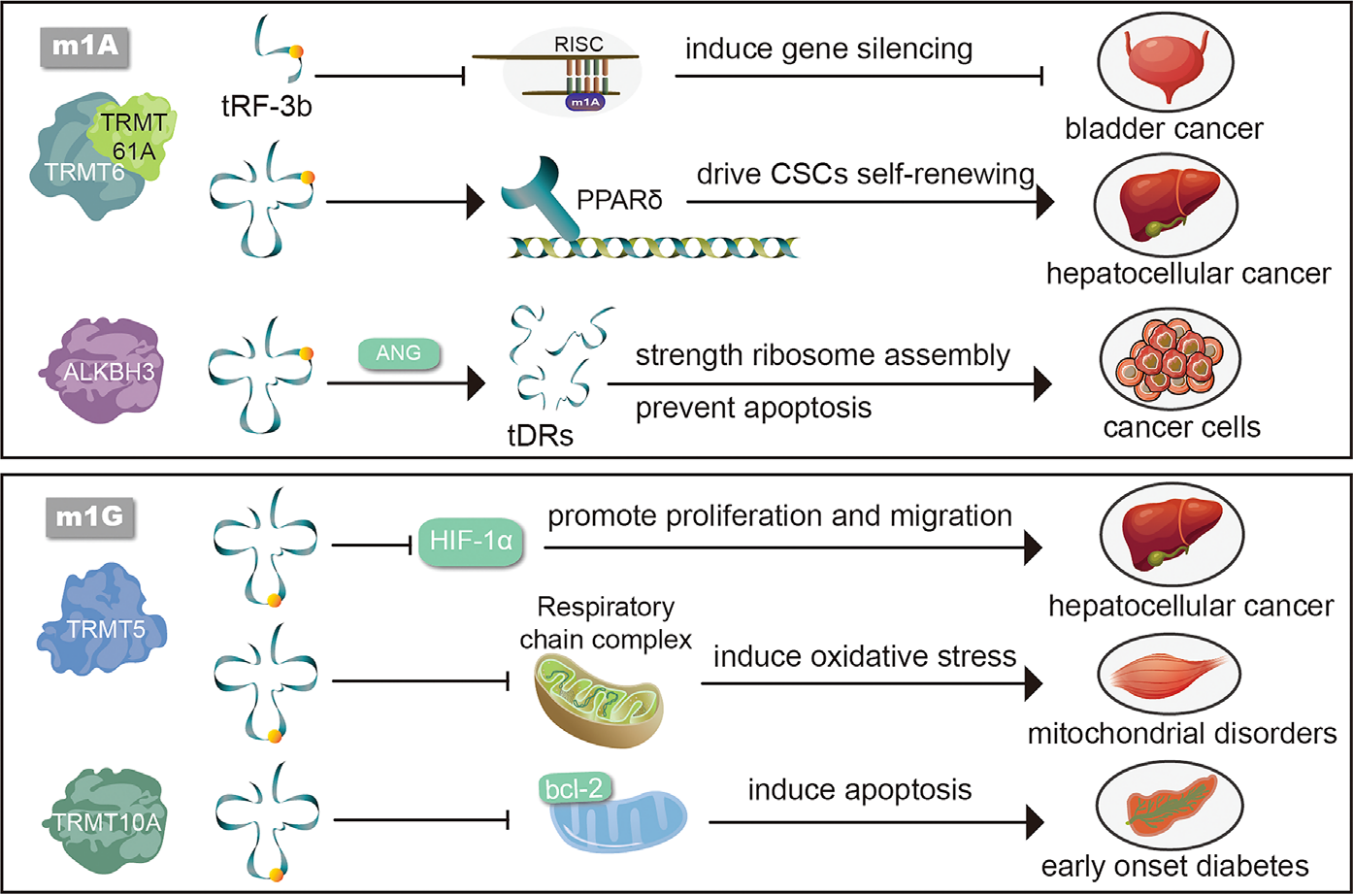
The key mechanisms of m1A and m1G modified tRNA in various diseases involve several important pathways. Overexpression of TRMT6/61A in neoplasm promotes tumour proliferation by up‐regulating m1A levels in tRFs in BLCA and stimulating PPARδ translation in HCC. ALKBH3 promotes cancer progression by inducing tDRs, which in turn enhances ribosome assembly and prevents apoptosis. TRMT5 serves as a target to inhibit the progression of HCC and mitochondrial disorder through separate mechanisms. This is achieved by inhibiting the HIF‐1α pathways and maintaining the function of the respiratory chain complex. Additionally, TRMT10A plays a role in preventing the early onset of diabetes by inhibiting Bcl‐2, thereby inhibiting apoptosis. tRF‐3b, tRNA‐derived fragments‐3b; RISC, RNA‐induced silencing complex; PPARδ, peroxisome proliferator‐activated receptor delta; CSC, cancer stem cells; ANG, angiogenin; tDRs, tRNA‐derived small RNAs; HIF‐1α, hypoxia inducible factor‐1α; Bcl‐2, B‐cell lymphoma‐2.

Various neoplastic diseases have been associated with the upregulation or overexpression of m1A related enzymes (Figure 2). In bladder cancer (BLCA) and hepatocellular carcinoma (HCC), the expression of TRMT6/61A is elevated, leading to increased m1A modification on tRNAs.^27^ In BLCA, higher m1A modification is observed on tRNA‐derived fragments (tRFs), which correlates with dysregulation of the tRF targetome.^27^, ^28^ In HCC, enhanced m1A methylation in tRNA promotes peroxisome proliferator‐activated receptor delta (PPARδ),^28^ triggering cholesterol synthesis, activating Hedgehog signalling, and ultimately driving the self‐renewal of liver cancer stem cells (CSCs)and tumorigenesis.^28^ In highly aggressive glioblastoma multiforme, unlike Grade II/III glioblastomas, a significant upregulation in TRMT6/TRMT61A expression was noted.^29^ Additionally, ALKBH3 promotes cancer proliferation, migration, and invasion by inducing tDRs and strengthening ribosome assembly, thereby facilitating cancer progression.^30^ Simultaneously, ALKBH3 prevents apoptosis induced by cytochrome c (Cyt c).^30^ Moreover, regulators including YTHDF1, TRMT61B, TRMT10C, and ALKBH1 are associated with immunotherapy response and prognosis in clear cell renal cell carcinoma (ccRCC).^31^

Studies have revealed that the m1A modification on tRNAs plays a role in neurological disorders and infectious diseases. Notably, adjustments in m1A level have been associated with efficient translation of MYC proteins in T cell activation and regulation of neuronal excitability in *Aplysia californica*.^32^, ^33^, ^34^ For instance, in patients with myoclonic epilepsy with ragged red fibres syndrome (MERRF), the absence of m1A58 in mt‐tRNA^Lys^ is attributed to the mitochondrial DNA mutation m.8344 A > G.^35^ In Alzheimer's disease, a study suggested that reduced m1A modification in tRNAs could potentially contribute to pathogenesis.^36^ Interestingly, m1A58 and TRMT6 are necessary for retrovirus replication.^37^ It is surprising that the RNA of HIV‐1 virions contains a significant amount of m1A, exclusively present in the tRNA.^38^ Experiments on T cell activation suggest that the overexpression of TRMT6/TRMT61A facilitates the transfer of m1A to a cluster of pre‐tRNA species, promoting proliferation of T cells and ensuring a prompt response of the adaptive immune system by enhancing MYC protein translation.^32^, ^33^

Furthermore, m1A in tRNA is hypothesized to be involved in the cellular response to oxidative stress.^39^ Studies suggest that oxidative stress leads to increased retention of m1A‐modified tRNAs in kidney cells.^40^ ALKBH8 functions by demethylating tRNA to modulate the generation of glutathione peroxidases (Gpx's), which are believed to play a protective role during APAP toxicity.^41^

### 2.2 N3‐Methylcytosine (m3C) modification in tRNA

m3C is primarily found in tRNA at position 32 in the anticodon loop, and it is also present at position 47:3 in the variable loop and position 20 in the D loop.^42^, ^43^ The methyltransferase‐like (METTL) family, which includes METTL2A/B, METTL6, and METTL8, serve as “writers” by adding a methyl group to the nitrogen atom at position 3 of cytosine (N3) at specific positions in tRNA.^44^ METTL2A/B is localized in the cytoplasm and is responsible for depositing m3C on tRNA(Thr)(UGU) and tRNA(Arg)(CCU), with G35 and U36 play an important role in substrate tRNA recognition.^44^, ^45^ DALR anticodon binding domain containing 3 (DALRD3) can form a complex with METTL2 to aid in the identification of specific tRNA^Arg^.^46^ METTL6 mainly localizes in the nucleus and promotes m3C formation in tRNA(Ser)(UGA/GCU),^45^, ^47^ a process that requires SerRS.^45^ METTL8 is predominantly found in the mitochondria and promotes m3C formation in mt‐tRNA(Ser)(UCN) and mt‐tRNA(Thr)(UCN).^48^ METTL8 recognizes the U34G35U36t6A37A38 motif in mt‐tRNA^Thr^ and requires 2‐Methylthio‐N 6‐prenyladenosine at position 37 for m3C modification of mt‐tRNA(Ser)(UCN).^49^ ALKBH3, located in the cytoplasm, acts as a demethylase for m3C in tRNA.^30^ However, the specific molecular mechanism underlying this process requires further investigation.

The m3C modification of tRNA plays a significant role in optimizing tRNA structural stability and regulating translation. Loss of METTL6 disrupts protein homeostasis and impairs translation processes.^50^ METTL8 is tightly associated with mitochondria, and its expression levels positively correlate with the activity of the mitochondrial respiratory chain.^48^ The absence of METTL8 leads to prolonged stalling of mitochondrial ribosomes at serine and threonine codons,^49^ possibly due to alterations in the migration patterns of mt‐tRNA (Ser)(UGA).^51^ While deletion of m3C does alter the structure of mt‐tRNAs, it does not influence their stability, implying that the effect of m3C on tRNA structure is more of a subtle adjustment rather than a mechanism to prevent misfolding.^43^, ^49^

The m3C modification of tRNA has significant implications for human diseases. METTL2A has been identified as a potential oncogene in breast cancer.^52^ METTL6 has been shown to promote the proliferation of HCC,^53^ and decreased expression of METTL6 can lead to a significant reduction in cell adhesion proteins, thereby inhibiting HCC cell proliferation, invasion, and migration.^54^ In lung cancer, the loss of METTL6 can reduce the sensitivity of lung cancer cells to cisplatin.^55^ Frameshift mutations in METTL8 in colorectal cancer may result in its inactivation, contributing to tumour progression.^56^ Upregulation of METTL8 has been observed in lung squamous cell carcinoma and highly aggressive pancreatic cancer,^48^, ^57^ which has a negative correlation with survival.^48^ Apart from the methyltransferase itself, deficiency of DALRD3 may promote developmental delays and progression of early‐onset epileptic encephalopathy by influencing METTL2‐mediated m3C modifications.^46^

Furthermore, evidence suggests that METTL6 and METTL8 play key roles in maintaining stem cell self‐renewal and pluripotency. METTL6 is involved in maintaining stem cell self‐renewal^53^; and its deficiency results in impaired pluripotent potential in mouse embryonic stem cells.^50^ Similarly, METTL8 supports the preservation of neural stem cells in both mice and the human cerebral cortex.^58^

### 2.3 N5‐Methylcytosine (m5C) modification in tRNA

m5C is commonly found in various RNAs, including mRNA and non‐coding RNA, predominantly deposited in tRNA and rRNA of eukaryotes.^59^ NSUN family 2/3/6 and DNMT2, also called tRNA Aspartic Acid Methyltransferase 1 or TRDMT1, are confirmed “writers” responsible for transferring methyl to the carbon‐5 position of cytosine at a specific site to form m5C.^60^, ^61^ NSUN2/3/6 utilize two cysteine residues at amino acid motifs VI and IV to form m5C,^62^ while DNMT2 only contains cysteine residues at motif VI.^63^ NSUN2 is primarily localized in the nucleus, where it catalyses methylation of pre‐tRNA(Gly)(GCC) methylation at C48‐50 in the variable loop, as well as methylates C34 in the anticodon loop and C48 of pre‐tRNA(Leu)(CAA).^64^, ^65^ Besides the cell nucleus, NSUN2 is also found in mitochondria in humans and mice, methylating C48‐50 of multiple tRNA.^66^ In contrast, NSUN3 is exclusively localized in mitochondria, where it methylates cytosine to m5C at C34 at the wobble position of the anticodon of mitochondria tRNA^Met^.^67^ NSUN6, a cytoplasmic protein enriched in the Golgi apparatus and pericentriolar matrix,^68^ specifically methylates C72 at the acceptor arm of tRNA(Thr)(TGT) and tRNA(Cys)(GCA).^68^ The CAA terminus is crucial for NSUN6 to methylate tRNA, accurately identified by NSUN6 via the PUA domain,^69^ indicating that the m5C modification of tRNA mediated by NSUN6 occurs at the late stage of tRNA biogenesis. DNMT2, localized in the nucleus, is specifically involved in generating m5C in tRNA(Asp)(GUC), tRNA(Gly)(GCC), and tRNA(Val)(AAC) at C38 in the anticodon loop.^70^ The “Erasers” majorly include the TET family and ALKBH1.^71^, ^72^ TET2 hydroxylates m5C to 5‐hydroxymethylcytosine (hm5C).^73^ In mitochondria, ALKBH1 utilizes iron and α‐ketoglutarate to oxidize m5C formed by NSUN3 to hm5C before further oxidizing hm5C to 5‐formylcytosine (f5C).^74^

In tRNA, accumulating evidence indicates that m5C plays a role in promoting tRNA structural stability and regulating protein synthesis.^75^ Notably, m5C is implicated in the interaction between Mg^2+^ and tRNA,^76^ enhancing the stability of tRNA tertiary structure.^77^ Moreover, m5C at position C48 stabilizes the “Levitt pair” formed between C48 and G15, a non‐canonical base pair that contributes to tRNA tertiary structure.^78^ Apart from its role in maintaining the tRNA stability, m5C can influence translation by forming in the anticodon loop, a process mediated by NSUN2, NSUN3, and DNMT2. In mitochondria, NSUN3‐mediated m5C is further oxidized to f5C by ALKBH1 at position 34 of tRNA (Met). This modification expands codon recognition by allowing AUA codons other than AUG codons during mitochondrial translation.^79^ Moreover, DNMT2‐mediated m5C at C38 contributes to the discrimination of near‐cognate codons for Asp, Val, and Gly. The presence of m5C at C38 enhances the ability of tRNA (Asp) to compete with near cognate tRNA at the ribosomal A‐site, ensuring the accurate incorporation of amino acids.^80^ In addition, DNMT2 facilitates the generation of charged tRNA^Asp^ through m5C generation, thereby enhancing the translation efficiency of proteins containing poly‐Asp sequences.^81^

**FIGURE 3:**
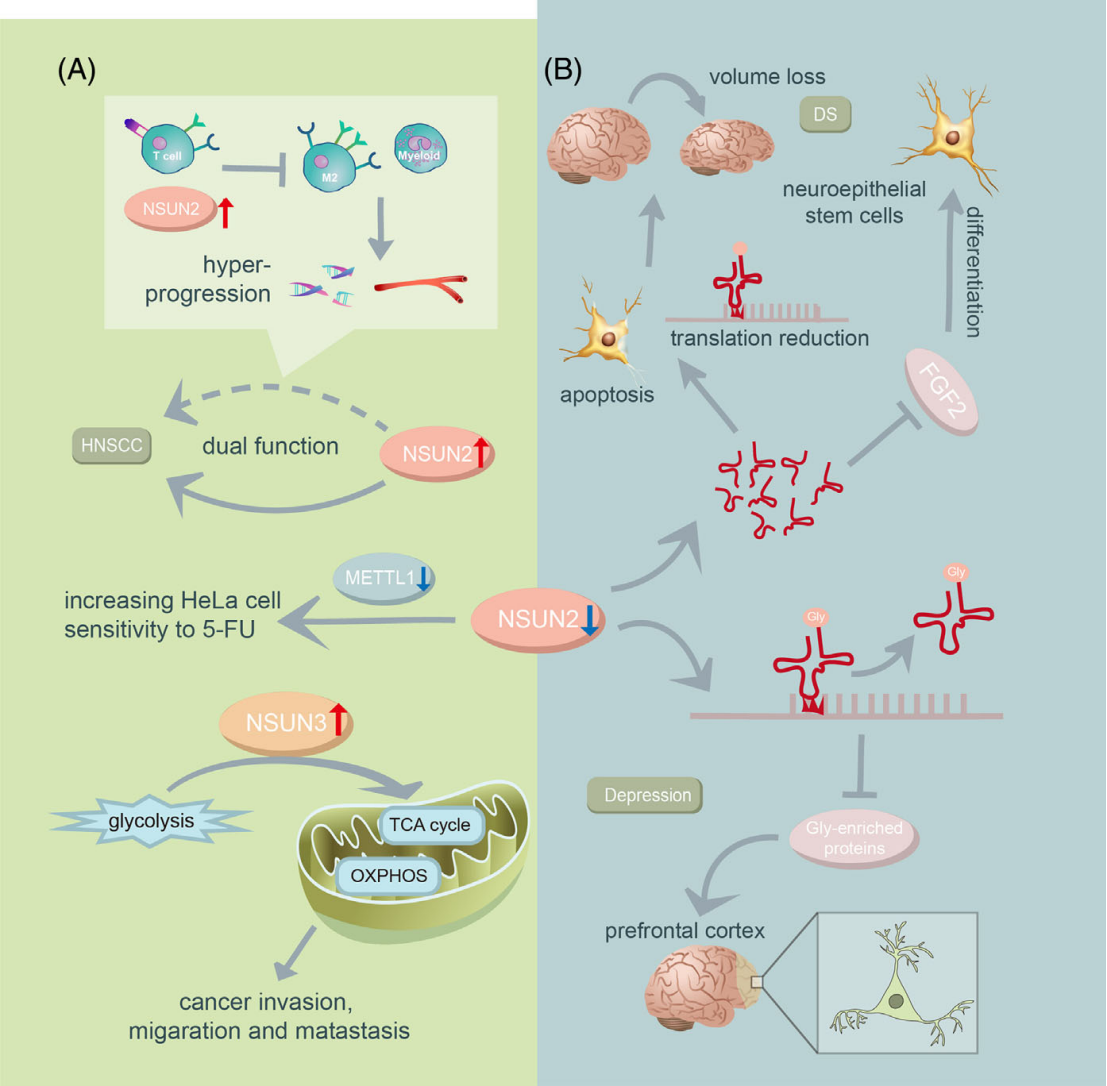
The molecular mechanisms of diseases involving m5C methyltransferases. (A) m5C methyltransferases and cancer. The expression level of NSUN2 plays a dual role in the prognosis of HNSCC. In human oral cancer cells, NSUN3 mediated m5C in mt‐tRNA induces cancer invasion, migration and metastasis by shifting glycolysis to oxidative phosphorylation (OXPHOS). Simultaneous knockdown of NSUN2 and METTL1 increases the sensitivity of HeLa cells to 5‐FU treatment. (B) Decreased NSUN2 expression is associated with nervous system disorders. The accumulation of tRFs due to NSUN2 deficiency triggers apoptosis and reduces the volume of neuronal cells in the cortex, hippocampus, and striatum by slowing down translation rates. Such accumulation also hinders the differentiation of neuroepithelial stem cells by impairing their responsiveness to FGF2. NSUN2 deficiency leads to a specific defect in the glycine codon, resulting in decreased levels of glycine‐enriched proteins. This disruption impairs synaptic signalling in pyramidal neurons in the prefrontal cortex, leading to deficits in contextual fear memory. DS, Dubowitz‐like syndrome; FGF2, fibroblast growth factor 2; Gly, glycine; HNSCC, head and neck squamous carcinoma; OXPHOS, oxidative phosphorylation; TCA cycle, tricarboxylic acid cycle.

The m5C modification of tRNA is intricately linked to tumorigenesis (Figure 3). NSUN3 and NSUN2 expressions are consistently upregulated in various tumour samples.^82^, ^83^ Notably, the expression level of NSUN2 positively associated with the unfavourable prognosis of head and neck squamous carcinoma (HNSCC).^84^ This correlation may stem from the interaction between NSUN2 and T cell activation status.^85^ In human oral cancer cells, NSUN3‐mediated m5C deficiency of mt‐tRNA does not influence primary tumour growth but impedes metastasis due to impaired mitochondrial metabolism.^86^ The collaborative action of NSUN2 and METTL1 shields Hela cells from 5‐FU‐induced cytotoxicity by maintaining tRNA stability, while not impacting the effects of paclitaxel and cisplatin.^87^ This suggests that combining m5C methylation inhibitors with chemotherapy drugs could improve the therapeutic benefit of chemotherapy in solid tumours.

Nervous system abnormalities are closely related to NSUN2‐mediated m5C in tRNA. NSUN2 is the first identified pathogenic gene linked to Dubowitz‐like syndrome (DS). Reduction in NSUN2 and m5C loss in tRNA (Asp)(GTC) at position 47/48 are observed in DS patients.^88^ The accumulation of tRFs due to NSUN2 deficiency triggers apoptosis and reduces the volume of neuronal cells in various brain regions by slowing down translation rates and activating stress pathways.^89^ This accumulation also hampers the differentiation of neuroepithelial stem cells, potentially due to impaired response to fibroblast growth factor 2 (FGF2).^90^ NSUN2 is also implicated in depression‐related behaviours.^91^ Reduced m5C level in tRNAs resulting from NSUN2 deficiency leads to a specific defect in the glycine codon, decreasing the levels of glycine‐enrich proteins. This alteration enhances glutamatergic neurotransmission, impairing synaptic signalling at pyramidal neurons in the prefrontal cortex and causing deficits in contextual fear memory.^91^

Apart from its role in disease progression, NSUN2‐mediated m5C modifications can also mitigate disease‐induced damage. A tRNA fragment (tRF‐Gln‐CTG‐026) generated from NSUN2 loss mitigates overall cellular protein synthesis by suppressing ribosome assembly, ultimately attenuating liver injury.^92^

### 2.4 N1‐methylguanosine (m1G) modification in tRNA

TRMT10A and TRMT5 are responsible for methylating m1G at positions 9 and 37 in tRNA, respectively.^93^, ^94^ In eukaryotes, TRMT5 plays a critical role in growth phenotype, mitochondrial protein synthesis, and function.^95^ Mitochondrial tRNAs modified with m1G could potentially contribute to mitochondrial genetic diseases, as defects in mitochondrial tRNAs frequently have significant implications.^96^ The deletion of TRMT10A leads to a reduction in m1G levels in tRNA and an increase in m1A levels in mRNA, indicating that TRMT10A plays a role in coordinating mRNA and tRNA methylation processes.^97^ Notably, AlkB is responsible for demethylating m1G, with the AlkB D15S mutant demonstrating significantly enhanced demethylation activity compared to the wild‐type enzyme.^98^ The presence of m1G and related enzymes in tRNA shows promise as biomarkers for carcinoma, early‐onset diabetes, and mitochondrial disorders. Understanding the roles of m1G modification and associated enzymes not only holds diagnostic and prognostic value but also offers potential avenues for improving treatment strategies in these conditions.

Significant differences in m1G9 levels have been observed between normal and tumour samples (Figure 2),^99^ indicating that m1G could serve as a promising biomarker to predict responses to cancer progression. In BLCA, a G‐type mismatch at the 9th position from tRNA^Glu^ with upregulated m1G in cell culture supernatant and urine has been detected. This corresponds to the known m1G site on parental tRNAs, and could potentially be investigated as a biomarker for BLCA detection or follow‐up.^100^, ^101^ In early‐stage breast cancer, the upregulation of m1G has been found to contribute to a nomogram prediction model.^102^ Additionally, hypoxia‐induced upregulation of m1G in ovarian cancers implies that the tumour microenvironment plays a role in cancer‐specific RNA modifications.^103^ Moreover, m1G has been implicated in HCC. In nonalcoholic fatty liver disease (NAFLD), m1G can participate in the pathogenesis confirmed by a fragment of U[m1G][m2G] significant upregulation.^104^ A recent study has highlighted that TRMT5 can suppress the HIF‐1α pathway, potentially inhibiting the progression of HCC.^105^ These findings underscore the importance of m1G and its regulatory mechanisms in various cancer types, pointing towards potential diagnostic, prognostic, and therapeutic implications in cancer research.

Mutations in methylases also play a role in the development of early‐onset diabetes and mitochondrial disorders. Deficiencies in TRMT10A and the resulting reduction in m1G levels have been associated with oxidative stress. In the pathogenesis of early‐onset diabetes, this reduction leads to apoptosis of pancreatic β‐cells, contributing to impaired glucose regulation.^106^ A report suggests higher murine atrial m1G levels indicate a potential tissue‐specific activity of TRMT10A in diabetes.^107^

Several case reports have highlighted that mutations in TRMT10A can lead to diabetes, autosomal‐recessive intellectual disability (ARID), microcephaly, and short stature.^108^, ^109^, ^110^, ^111^, ^112^, ^113^ On the other hand, mutations in TRMT5 that cause hypomodification of m1G are linked to deficiencies in respiratory‐chain complexes, particularly in skeletal tissues, resulting in various mitochondrial disorders.^114^ These disorders include complex hereditary spastic paraparesis, combined oxidative phosphorylation deficiency 26 (COXPD26), demyelinating peripheral neuropathy, and non‐cirrhotic portal hypertension (NCPH).^115^, ^116^, ^117^, ^118^ Besides, mutations in mt‐tRNA introducing TRMT5‐catalysed m1G37 modification have been correlated with hypertension and deafness.^119^, ^120^ TRMT5 can act as a reliable gene signature to promote early detection of Ischemic cardiomyopathy (ICM).^121^ These findings underscore the critical role of TRMT10A and TRMT5 mutations in the pathogenesis of early‐onset diabetes and various mitochondrial disorders, highlighting their relevance in clinical diagnosis and management strategies.

### 2.5 N2‐methylguanosine (m2G) modification in tRNA

m2G and its derivatives, primarily m2, 2G, are typically located at positions G6 and G26 in tRNA. These modified nucleosides exhibit a conformational preference in the tRNA hinge region at the 26th position, influencing base‐stacking interactions crucial for protein biosynthesis.^122^ In humans, three methyltransferases are known to be involved in m2G/m2,2G26 modifications: TRMT1, TRMT112/TRMT11, and TRMT112/THUMPD3, TRMT1 is responsible for the independent methylation of m2G26 or m2,2G26 in tRNA.^123^ On the other hand, TRMT112/TRMT11 and TRMT112/THUMPD3 methylate m2G10 and m2G6/7, respectively.^124^ A study focusing on 26 cytoplasmic tRNAs highlighted the importance of the characteristic 3′‐CCA sequence for TRMT112/THUMPD3 in recognizing and modifying tRNAs with m2G6 and 7.^125^ Cells lacking THUMPD3 showed impaired global protein synthesis and reduced growth.^125^ Regarding demethylation, the AlkB D135S/L118V mutant has been found to efficiently convert m2,2G into m2G with specificity.^126^ Additionally, DNMT2 also plays a role in the m2G modification, although the exact mechanisms remain unclear. Deletion of DNMT2 altered the small RNA expression profile in sperm, leading to changes in levels of tsRNA with increased m2G, suggesting a potential role in paternal epigenetic memory.^127^ These findings shed light on the intricate regulatory mechanisms governing tRNA modifications and their impact on protein synthesis and epigenetic processes.

The absence of m2G and m2,2G nucleoside modifications can cause alterations in global protein synthesis, redox homeostasis, and growth. TRMT1 down‐regulation decreases proliferation rates, changes global protein synthesis, and perturbates redox homeostasis by increasing endogenous reactive oxygen species (ROS) levels and developing hypersensitivity to oxidizing agents.^128^ This highlights the important role of m2,2G in redox homeostasis. Enzymatic modifications in tRNA by TRMT1 and DNMT2 enzymes may promote the development and transmission of carcinoma, lipid metabolic disorders, and ID. In tumorigenesis, TRMT1 promotes radio‐resistance in breast cancer.^129^ As mentioned above, a fragment of U[m1G][m2G] is significantly upregulated in HCC and NAFLD.^104^ Deletion of DNMT2 in mouse completely removes the high‐fat‐diet‐induced metabolic disorders transmission to offspring through sperm small non‐coding RNA (sncRNA). This is achieved by preventing an increase of m5C and m2G in the 30–40 nt RNA fractions of sperm.^127^ Lack of m2,2G has key perturbations in cellular homeostasis.^128^ An intellectual disability (ID)‐associated missense variant in TRMT1 has been identified to impair tRNA modification by altering enzymatic activity and converting an arginine residue to cysteine within the methyltransferase domain, resulting in a deficiency of m2,2G within tRNAs.^130^ Additionally, TRMT1 cleavage by the SARS‐CoV‐2 virus has significant implications for viral pathogenesis. Recent studies have demonstrated that the SARS‐CoV‐2 Main Protease Nsp5 can recognize and cleave TRMT1,^131^, ^132^ potentially influencing viral pathogenesis by influencing protein translation and oxidative stress response.^131^, ^132^ Furthermore, TRMT1 has been linked to kidney renal clear cell carcinoma (KIRC) relevant to SARS‐CoV‐2 infection, suggesting its potential as a reliable prognostic predictor and a promising therapeutic target for KIRC and COVID‐19.^133^ These findings underscore the intricate interplay between tRNA modifications, disease states, and viral infections, highlighting the importance of further research in this area for both understanding disease mechanisms and developing potential therapeutic interventions.

### 2.6 N7‐methylguanosine (m7G) modification in tRNA

**FIGURE 4:**
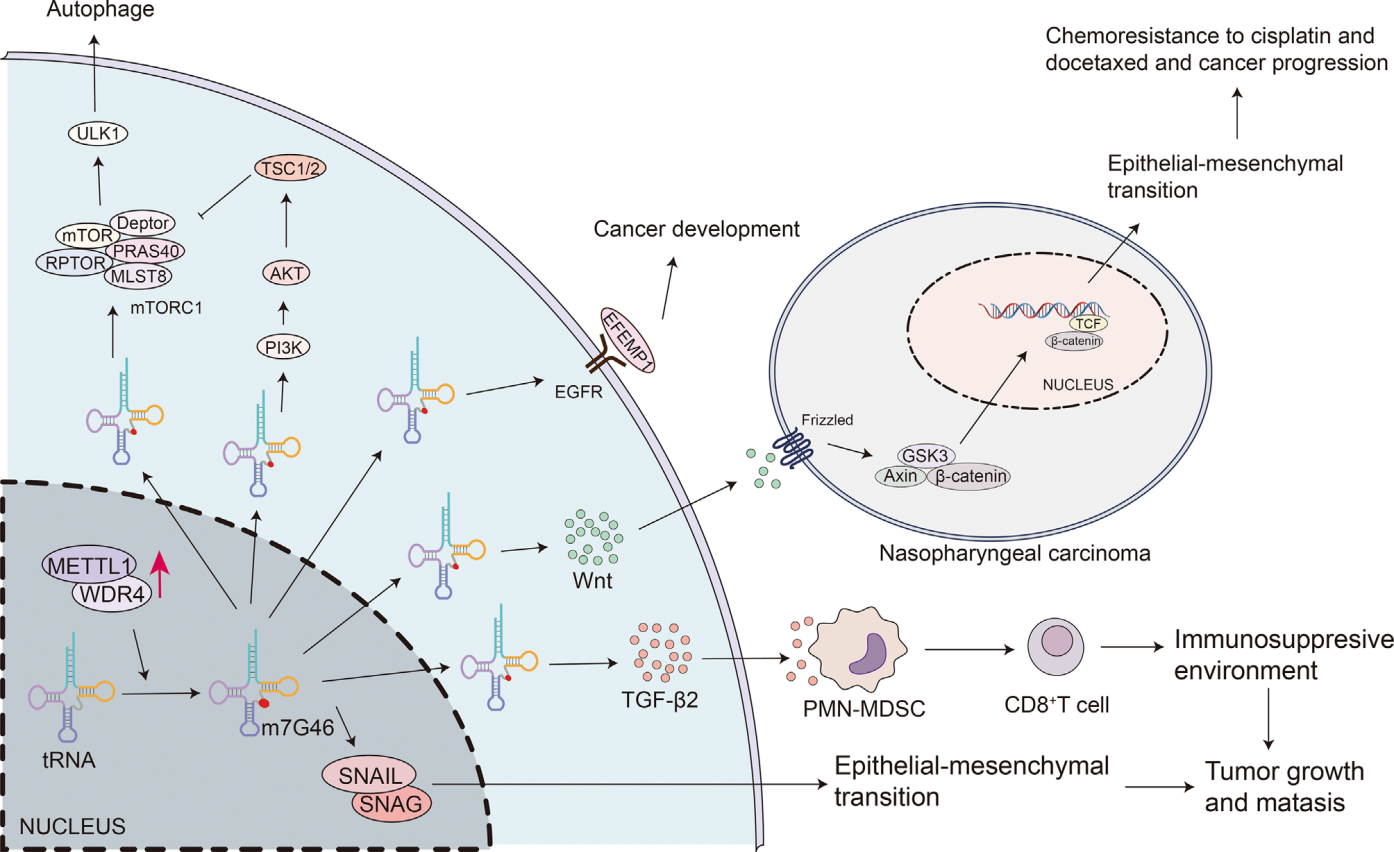
The molecular mechanisms of carcinoma involving m7G46. m7G46 is the most extensively investigated tRNA methylation regulated by METTL1/WDR4. m7G46 regulates the classic pathways PI3K/AKT/mTOR and EGFR to induce autophagy and cancer development. Moreover, via the SLUG/SNAIL pathway, m7G46 stimulates the recruitment of CD8 + T cells triggering epithelial‐mesenchymal transition (EMT), which promotes tumour growth and metastasis. Moreover, by regulating the Wnt pathway, m7G46 activates EMT and cancer progression in nasopharyngeal carcinoma.

The m7G modification is commonly found in in mRNA, tRNA, and rRNA. In tRNA, METTL1/WDR4 (WD Repeat Domain 4) mediates the modification of m7G, with a dominant modification at position G46 (m7G46) in the variable loop is dominant in a large subset. The N terminus of METTL1 activates the methylation of m7G by coupling with a cofactor that influences tRNA conformational changes.^134^ Furthermore, the tRNA central structure core is stabilized by m7G46‐C13‐G22 forming a tertiary base pair with m7G46.^135^ In humans, m7G modification plays a role in promoting induced pluripotent stem cell (iPSC) differentiation into endothelial progenitor cell (EPC). In embryonic stem cells (mESCs), m7G in tRNA is essential for the proper translation of genes related to the cell cycle and brain malformation.^136^ In recent years, m7G in tRNA has gained significant attention in the medical field, particularly in oncology. The presence of m7G‐modified tRNAs significantly increases in cancers, leading to enhanced translation of mRNAs. This suggests that m7G remodels the mRNA translatome to drive oncogenic transformation.^137^ Targeting m7G could potentially serve as an anti‐cancer strategy. This review explores several cancers with significantly upregulated m7G modification in tRNA and overexpression of METTL1/WDR4, elucidating the underlying mechanisms (Figure 4).

The METTL1/WDR4‐mediated m7G modification in tRNA plays a pivotal role in various digestive system neoplasms, including HCC, oesophageal squamous cell carcinoma (ESCC), gastric cancer (GC), intrahepatic cholangiocarcinoma (ICC). In HCC, the overexpression of METTL1/WDR4 is associated with advanced‐stage tumours and a poor prognosis. Studies have indicated that silencing METTL1/WDR4 suppresses HCC progression, while its expression promotes tumour proliferation, migration, and invasion.^138^ Upregulation of METTL1/WDR4 leads to resistance to Lenvatinib in HCC, providing a promising approach to overcome this resistance.^139^ Besides chemotherapy, high METTL1 expression in HCC is linked to poor prognosis following radiotherapy.^140^ Research has demonstrated significant upregulation of METTL1 in cases of insufficient radiofrequency ablation (iRFA) recurrent HCC, involving different pathways. Recurrence after iRFA has been linked to the TGF‐β pathway. Strategies such as TGF‐β signalling blockade, knockdown of hepatoma‐intrinsic Mettl1 or Tgfb2, or interruption of the METTL1‐TGF‐β2‐PMN‐MDSC axis can mitigate tumour progression induced by iRFA and restore the CD8+ T cell population.^141^ Furthermore, upregulation of m7G has been shown to enhance SLUG/SNAIL translation, thereby restoring malignant potential following iRFA.^142^

In GC, lower m7G levels correlate with fewer immune escape, more immune cell infiltration, better clinical stage, and longer survival time, resulting in a better efficacy of immunotherapy.^143^ In contrast, higher m7G levels may cause poorer prognosis.^143^ A m7G‐related prognostic model was established, which demonstrated excellent prognostic discrimination, which can act as an important tool for guiding personalized treatment and forecasting prognosis in GC patients.^144^ In ESCC tissues, METTL1/WDR4 is significantly overexpressed and generally linked to poor prognosis.^145^ m7G in tRNA promotes ESCC tumorigenesis via the RPTOR‐ULK1‐autophagy axis. Targeting METTL1/WDR4 reduces m7G modification and subsequently decreases the translation of certain oncogenic transcripts, including Regulatory Associated Protein of mTOR Complex 1 (PRTOR).^146^ This reduction in RPTOR translation causes ULK1 dephosphorylation, which hyperactivates MTORC1‐mediated autophagy, ultimately causing cell death in ESCC.^146^ In intrahepatic cholangiocarcinoma (ICC), there is a significant METTL1/WDR4 upregulation, which is strongly linked to poor prognosis.^147^ In prostate tumours, METTL1 is also overexpressed both in primary and advanced stages, suppressing immune effectors, interferon pathways, and tumour growth suppression.^148^

Besides, METTL1/WDR4 is significantly upregulated and correlates with poor prognosis in nasopharyngeal carcinoma (NPC). The overexpression of METTL1/WDR4 in NPC promotes growth, metastasis, and chemoresistance to cisplatin and docetaxel.^149^ This upregulation of METTL1/WDR4 leads to the activation of the WNT‐β‐catenin signalling pathway through the transcription factor ARNT, thereby facilitating epithelial‐mesenchymal transition (EMT).^149^ In human lung cancer, elevated levels of METTL1/WDR4 expression levels are also associated with a poor prognosis. Depletion of METTL1/WDR4 disrupts m7G tRNA modification, resulting in reduced colony formation, cell invasion, proliferation, and impaired tumorigenic capacities.^150^

Through its control of global mRNA translation, including the PI3K‐AKT–mTOR signalling pathway, METTL1 has been identified as a promoter of the development and malignancy of HNSCC.^151^ Furthermore, alterations in the immune landscape have been observed as a result of METTL1 activity in this context.^151^ In advanced neuroblastoma, METTL1 is associated with poor prognosis and independently acts as a risk factor.^152^ Notably, the METTL1‐m7G‐EGFR/EFEMP1 axis plays an important role in the development of BLCA.^153^ A novel RNA epigenetic mechanism has unveiled that dual m6A/m7G RNA modifications mediated by METTL3/METTL1 enhance TROP2 translation, thereby promoting BLCA progression.^154^ The METTL1/WDR4‐mediated m7G in tRNA influences oncogenic mRNA translation, which is pivotal in driving osteosarcoma advancement and resistance to doxorubicin chemotherapy.^155^

In addition to promoting tumorigenesis, knockout of METTL1 or WDR4 results in impaired neural lineage differentiation and mESC self‐renewal.^136^ Specifically, METTL1 depletion leads to a neurodevelopmental disorder by inhibiting neuroectoderm and promoting mesoderm differentiation.^156^ Mutations in the WDR4 gene mutation can lead to microcephalic primordial dwarfism through amino acid substitution that disrupt the m7G46 modification in tRNA.^157^ Apart from its role in the nervous systems, recent research has identified WDR4 expression as a significant hub gene in the context of osteoarthritis, showing significant upregulation in osteoarthritis tissues.^158^

### 2.7 N5‐methyluridine (m5U) modification in tRNA

The m5U54 modification of tRNAs in the cytoplasm is a widely observed and evolutionarily conserved tRNA modification across various species. In mammals, TRMT2A functions as the methyltransferase responsible for m5U54 in cytoplasmic tRNAs, while TRMT2B methylates m5U54 in both cytoplasmic and mitochondrial tRNAs.^159^, ^160^ This modification plays a crucial role in stabilizing the tRNA structure and regulating cell proliferation. m5U54 can stabilize the tRNA structure and regulate cell proliferation. m5U54 acts as a protective mark against tRNA cleavage by promoting the stability of the L‐shaped tRNA structure through the formation of a reverse Hoogsteen base pair with A58.^161^ Hypomodification of m5U54 by TRMT2A leads to a reduction in m5U54 levels in tRNA, resulting in the overexpression of the ribonuclease angiogenin (ANG). This, in turn, generates 5’ tRNA‐derived stress‐inducible RNAs associated with RNA stability and cellular stress response.^162^ Moreover, the methyltransferases might have double roles in tRNA maturation and cell viability beyond their methylation catalysis functions.^163^, ^164^

Research on m5U in disease is limited, but studies have shown that NSUN2 deficiency leads to decreased levels of m5U and m5C modifications on tRNAs, leading to the production of various tsRNAs, particularly Class I tsRNAs or tRF‐1 s. Specific tsRNAs like tRF‐Gln‐CTG‐026 have been shown to modulate interactions within the ribosome and global protein synthesis, thereby mitigating liver injury.^92^ Furthermore, modifications like m5U on tRNA^Ile^ have been found to enhance cytotoxicity against cancer cells.^165^ This effect was attributed to the improved stability of the tertiary structure of the 3′‐t‐half mimic.^165^ The overexpression of TRMT2A has been linked to decrease in cell proliferation and changes in DNA content, suggesting its potential role as a regulator of cell cycle.^166^ Recent research established a transfer learning deep neural network RNADSN to enhance the accuracy of predicting m5U modifications by learning the similarities between tRNA and mRNA m5U.^167^ These findings offer promising insights for targeted therapies in the future.

### 2.8 2‐O‐methylation (Nm) in tRNA

2‐O‐methylation, also known as Nm, is a widely observed modifications in RNA. The 18th position of guanosine (Gm18) has a close association with immune response. Gm18 is known to enhance the immune response and serves as a universal regulator in humans, specifically inhibiting innate immune activation triggered by RNA. In particular, Gm18 suppresses the production of IFN‐α through Toll‐like receptors 7 (TLR7) in plasmacytoid dendritic cells (pDCs) by impairing key signalling pathways such as NF‐κB and MAP kinase activation. It also competes with stimulatory RNA for receptor binding.^168^ The absence of Gm18 can alter the immune stimulatory properties of human tRNA, leading to increased immunostimulation of peripheral blood mononuclear cells, which has the potential to contribute to the development of autoimmune diseases.^169^ On the other hand, the double methylation of tRNA‐U54 to 2‐O‐methylthymidine (Tm) works synergistically to reduce the immune response mediated by TLR7.^170^

The prevalence of Nm in the tRNA^Phe^ anticodon loop suggests a regulatory role in human neuronal development.^171^ WDR6 is essential for human tRNA^Phe^, along with FTSJ1, and THADA. Mutations in FTSJ1 are implicated in non‐syndromic X‐linked intellectual disability (NSXLID), where Gm34 may serve as a critical modification.^172^ In ftsj1 knockout cells, there is a reduction in the translation efficiency of the UUU codon.^173^ Moreover, approximately 40% of the genes showing a bias towards high TTT are associated with brain or nervous functions.^173^ In Ftsj1 KO mice, there is a specific decrease in the steady‐state level of tRNA^Phe^ in the brain, leading to a slower decoding process at Phe codons^174^ Consequently, these mice exhibit immature synaptic morphology and abnormal synaptic plasticity,^174^ which are associated with anxiety‐like behaviours and memory deficits.^174^ The absence of Nm in the anticodon loop of tRNA triggers the activation of the general amino acid control pathway (GAAC), a pathway that is highly conserved across different eukaryotic organisms and may play a role in FTSJ1‐mediated NSXLID.^175^

## 3 APPLICATION OF TARGETING tRNA METHYLATION IN THERAPY

Inhibiting or activating the enzymes involved in tRNA methylation can significantly impact the deposition of methylation modifications in tRNAs. Given the critical role of tRNA methylation in embryonic development, tissue differentiation, and disease progression, there is a growing interest in developing drugs that target these modifications.

Currently, drug design efforts predominantly focus on inhibiting the activity of enzymes involved in tRNA methylation. However, some enzyme activators have also shown promise in treating various diseases (Table 2). While all inhibitors and activators targeting tRNA methylation are still in the laboratory stage, there is a belief that leveraging inhibition or activation of tRNA methylation for clinical treatments will emerge as a prominent trend in the future.

**TABLE 2:** The summary of different types of drugs targeting unfolded protein response.

Class	Compound	Target	IC50 or EC50/μM	Mechanism	Effect	References
Methyltransferases inhibitor	Quercetin	METTL3	2.73	Competitive inhibitor	Reduces m6A level and inhibits the proliferation of breast cancer cells and liver cancer cells	176
	STM2457	METTL3	0.0169	Competitive inhibitor	Reduces m6A level, inhibits the proliferation of AML cells and enhances apoptosis and differentiation	177
	UZH1a	METTL3	0.28	Competitive inhibitor	Reduces m6A level of AML and osteosarcoma cells	178
	UZH2	METTL3	0.005	Competitive inhibitor	Reduces m6A level in AML and prostate cancer cells	179
	Cpd‐564	METTL3	NA	Competitive inhibitor	Reduces m6A level and attenuates AKI	180
	CDIBA	METTL3	2.81	Allosteric inhibitor	Reduces m6A level and inhibits the proliferation of AML cells	181
	Eltrombopag	METTL3	4.55	Allosteric inhibitor	Reduces m6A level and inhibits the proliferation of AML cells	182
	Azacytidine	DNMT2	NA	Incorporating nascent tRNAs	Inhibits m5C deposition at C38 of tRNA(Asp)	183
Methyltransferases activator	methyl piperazine‐2‐carboxylate	METTL3	0.0125	Binding to the METTL3‐14‐WTAP Complex active site	Increases m6A	220
Demethylases inhibitor	Rhein	FTO/ALKBH2/ALKBH3	21/NA/5.3	Competitive inhibitor	Increases m6A, m1A and/or m3C levels and inhibits cells proliferation	185, 186
	Meclofenamic acid	FTO	12.5	Competitive inhibitor	Increases m6A level	192
	Entacapone	FTO	3.5	Competitive inhibitor	Increases m6A level, reduces fasting blood glucose concentration and leading to weight loss	194
	Fluorescein Derivatives	FTO	1.0–7.0	Competitive inhibitor	Increases m6A level and marking FTO	193
	FTO‐04	FTO	3.39	Competitive inhibitor	Increases m6A level and inhibits glioblastoma stem cells proliferation	223
	FTO‐43	FTO	1.0	Competitive inhibitor	Increases m6A level and inhibits the proliferation of various cancer cells	196
	Saikosaponin‐d	FTO	0.46	Competitive inhibitor	Increases m6A level, inhibits AML cells proliferation, promotes apoptosis and cell‐cycle arrest, and prevents resistance to tyrosine kinase inhibitors	199
	4‐amino‐8‐chloroquinoline‐3‐carboxylic acid/8‐aminoquinoline‐3‐carboxylic acid	FTO	1.46/28.9	Competitive inhibitor	Increases m6A level and promotes survival of dopamine neurons	195
	CHTB	FTO	39.24	Competitive inhibitor	Increases m6A level	188
	Nafamostat mesylate	FTO	13.77	Competitive inhibitor	Inhibits FTO	192
	Clausine E	FTO	27.79	Competitive inhibitor	Inhibits FTO	191
	Radicicol	FTO	16.04	Competitive inhibitor	Inhibits FTO	189
	N‐CDPCB	FTO	4.95	Competitive inhibitor	Increases m6A level	190
	CS1 and CS2	FTO	0.1426/0.7128	Competitive inhibitor	Increases m6A level, inhibits AML cells differentiation and viability, and inhibiting leukaemia stem self‐renewal	201
	FB‐23 and FB23‐2	FTO	0.06/2.6	Competitive inhibitor	Increases m6A level, inhibits AML cells proliferation, promotes AML cells apoptosis and myeloid differentiation	200
	Dac51	FTO	0.4	Competitive inhibitor	Increases m6A level, inhibits tumour growth, and promotes T cells infiltration	202
	R‐2‐hydroxyglutarate	FTO	133.3	Competitive inhibitor	Increases m6A level, inhibits the proliferation of various cancer cells	197
	18,097	FTO	0.64	Competitive inhibitor	Increases m6A level and inhibits metastasis, growth and lipogenesis of breast cancer cells	198
	ALK‐4	ALKBH5	NA	NA	Increases m6A level and reducing suppressive immune cells infiltration	207
	IOX1	ALKBH5	NA	Competitive inhibitor	Increases m6A level and lessening AKI	208, 224
	2‐((1‐hydroxy‐2‐oxo‐2‐phenylethyl)sulfanyl)acetic acid/4‐(((furan‐2‐yl)methyl)amino)‐1,2‐diazinane‐3,6‐dione	ALKBH5	0.84/1.79	Competitive inhibitor	Inhibits AML cells viability	204
	20 m	ALKBH5	0.021	Competitive inhibitor	Inhibits m6A demethylation and stabilizes the ALKBH5 structure	203
	MV1035	ALKBH5	NA	Competitive inhibitor	Increases m6A level, inhibits migration and invasion of glioblastoma, and overcoming drug resistance	205, 206
	Indenone derivatives	ALKBH3	9.8	Competitive inhibitor	Inhibits lung carcinoma cells proliferation and increases MMS sensitivity	209
	HUHS015	ALKBH3	0.67	Competitive inhibitor	Inhibits prostate cancer cells proliferation	210
	1‐(5‐fluoro‐1H‐benzimidazol‐2‐yl)‐3‐methyl‐4‐phenyl‐1H‐pyrazol‐5‐ol	ALKBH3	2.9	Competitive inhibitor	Inhibits prostate cancer cells proliferation	211
	Bobcat339	TET1 and TET2	33/73	Competitive inhibitor	Decreases global 5hmC levels	212
Binding protein inhibitors	AVJ16	IGF2BP1	NA	Competitive inhibitor	Inhibits migration of non‐small‐cell lung cancer cells	216
	BTYNB	IGF2BP1	5.0	Competitive inhibitor	Inhibits the proliferation of IGF2BP1‐positive cells and cancer growth	217, 218
	Cucurbitacin B	IGF2BP1	NA	Allosteric inhibitor	Promotes immune cell infiltration and suppresses PD‐L1 expression	219

Abbreviations: 20 m, 5‐hydroxy‐1‐(3‐(trifluoromethyl)phenyl)‐1 H‐pyrazole‐3‐carboxylic acid; AKI, acute kidney injury; ALKBH2/3, alkylation repair homologue 2/3; AML, acute myeloid leukaemia; DNMT2, DNA methyltransferase 2; FTO, fat mass and obesity‐associated gene; m3C, 3‐methylcytosine; m5C, 5‐methylcytosine; m6A, N(6)‐methyladenosine; METTL3, methyltransferase‐like family 3.

The potential to modulate tRNA methylation through enzyme inhibition or activation opens up new avenues for therapeutic interventions. As research in this area progresses, it is anticipated that novel drug candidates targeting tRNA methylation modifications will be developed, offering innovative approaches to address a wide range of diseases and medical conditions.

### 3.1 Inhibition of methyltransferases activity

Several compounds can inhibit the activity of METTL3. Quercetin, a natural small molecular compound, can stably bind to the METTL3 pocket of the adenosine moiety of SAM thereby suppressing the activity of METTL3.^176^ In breast and liver cancer cells, quercetin treatment inhibits the proliferation of tumour cells.^176^ STM2457, a potent and selective METTL3 inhibitor, precisely targets the SAM binding site, effectively suppressing m6A levels without impacting other modifications. Intriguingly, STM2457 delays AML cell proliferation while promoting apoptosis and differentiation, sparing normal haematopoietic function.^177^ UZH1a and quercetin share the same binding site. It can decrease m6A level in AML cells, osteosarcoma cells, and embryonic kidney cells, without affecting other modifications.^178^ In AML and prostate cancer cells, UZH2 can selectively inhibit METTL3.^179^ Cpd‐564 has been shown to bind the catalytic domain which decreases METTL3 activity. It can attenuate acute kidney injury caused by cisplatin in mice by downregulating the m6A levels.^180^ Apart from inhibitors that directly bind to the active pocket of SAM, several compounds have been designed to act as allosteric inhibitors of METTL3. 4‐[2‐[5‐chloro‐1‐(diphenylmethyl)‐2‐methyl‐1H‐indol‐3‐yl]‐ethoxy]benzoic acid (CDIBA) and eltrombopag are METTL3 allosteric inhibitors which inhibit the proliferation of AML cells.^181^, ^182^

In addition to METTL3, a few inhibitors for other transferases have been identified. Azacytidine can inhibit DNMT2‐mediated m5C in tRNA, thereby suppressing tumour proliferation.^183^ The nitrogen in azacytidine can replace the 5‐carbon atom of cytidine in nascent tRNA without altering the covalent binding of DNMT2 to the target site, thereby suppressing the function of DNMT2.^184^

### 3.2 Inhibition of demethylases activity

In contrast to methyltransferases, demethylase inhibition elevates intracellular methylation levels, thereby promoting disease progression. Notably, FTO, ALKB homologue, and TET inhibitors have demonstrated efficacy in suppressing cancer cell proliferation and enhancing therapeutic outcomes. Rhein is the first FTO competitive inhibitor to be discovered with low cytotoxicity.^185^ However, rhein has low specificity, which also inhibits ALKBH2 and ALKBH3 leading to the inhibition of the proliferation of glioblastoma multiforme cells.^186^ Notably, rhein inhibits FTO and ALKB by binding to different sites. Rhein binds to m6A site of FTO while 2‐oxoglutarate (2OG) binds to the binding site of ALKB.^185^, ^186^ Besides rhein, more potent competitive inhibitors that are highly selective for FTO have been developed, which inhibit FTO by binding to the m6A site. Meclofenamic acid,^187^ CHTB,^188^ Radicicol,^189^ N‐CDPCB,^190^ Clausine E,^191^ and nafamostat mesylate have the potential to inhibit FTO activity in a dose‐dependent manner but their effects on disease have not been clarified. Several fluorescein derivatives can inhibit FTO activity while labeling FTO.^192^, ^193^ As an obesity‐related protein, inhibition of FTO by entacapone can reduce the weight and fasting blood glucose concentration of mice.^194^ Studies have shown that 4‐amino‐8‐chloroquinoline‐3‐carboxylic acid and 8‐aminoquinoline‐3‐carboxylic acid can prevent apoptosis of dopamine neurons induced by growth factor deprivation and promote their survival, thereby ameliorating neurodegenerative diseases.^195^ FTO inhibitors can treat various cancers. FTO‐43 and oncometabolite R‐2‐hydroxyglutarate reported to suppress the proliferation of various cancer cells. On the hand, 18,097 can inhibit the lipogenesis, proliferation and metastasis of breast cancer cells in vivo and in vitro.^196^, ^197^, ^198^ In AML cells, Saikosaponin‐d,^199^ FB‐23, FB23‐2,^200^ CS1 and CS2 hindered the proliferation, promoted the differentiation and apoptosis, and exerted strong antitumor activity. Beside this, Saikosaponin‐d can overcome resistance to tyrosine kinase inhibitors.^199^, ^201^ CS1 and CS2 prevented immune escape of AML cells by increasing the sensitivity of cells to CD8^+^ T cells.^201^ Similar to CS1 and CS2, Dac51 inhibits tumour growth by promoting T cell infiltration into tumour microenvironment (TME) by inhibiting FTO.^202^

For the ALKB homologue, some competitive inhibitors of ALKBH5 and ALKBH3 have been developed. 5‐hydroxy‐1‐(3‐(trifluoromethyl)phenyl)‐1H‐pyrazole‐3‐carboxylic acid (20 m) is one of the ALKBH5 inhibitors known to inhibit m6A demethylation and stabilize the ALKBH5 structure.^203^ 2‐((1‐hydroxy‐2‐oxo‐2‐phenylethyl)sulfanyl)acetic acid and 4‐(((furan‐2‐yl)methyl)amino)‐1,2‐diazinane‐3,6‐dione are potent inhibitors of the viability of AML cells.^204^ MV1035 decreases the migration and invasion of cells and overcomes temozolomide resistance in glioblastoma by inhibiting ALKBH5. Notably, IOX1 and ALK‐4, both of which are ALKBH5 inhibitors, exert opposite effects via inhibiting ALKBH5.^205^, ^206^ The combination of ALK‐4 and GVAX with PD‐1 Ab was reported to enhance the efficacy of immunotherapy in melanoma and colorectal cancer by inhibiting Tregs and myeloid derived suppressor cells infiltrating the TME.^207^ However, IOX1 attenuates AKI by promoting Tregs recruitment and reducing the number of neutrophils and macrophages.^208^ Currently, indenone derivatives,^209^ HUHS015 and 1‐(5‐fluoro‐1H‐benzimidazol‐2‐yl)‐3‐methyl‐4‐phenyl‐1H‐pyrazol‐5‐ol (compound 7I) are the known inhibitors of ALKBH3.^210^, ^211^ Compound 5c which is derived from indenone has been shown to inhibit lung carcinoma cells proliferation and increase sensitivity to methyl methane sulfonate (MMS) by blocking ALKBH3.^209^ HUHS015 can strongly inhibit the proliferation of prostate cancer cells, but it is easily broken down in vivo.^210^ Therefore, 1‐(5‐fluoro‐1H‐benzimidazol‐2‐yl)‐3‐methyl‐4‐phenyl‐1H‐pyrazol‐5‐ol (compound 7I) was designed based on HUHS015. Apart from inhibiting the growth of prostate cancer, compound 7I exhibits higher stability and can maintain a higher blood concentration in the body.^211^

Bobcat339 is the first identified small molecule inhibitor of TETs that reduces global hm5C levels.^212^ However, studies have shown that at the reported concentrations, Bobcat339 cannot inhibit TETs effectively, and its activity can be increased by Cu^2+^.^213^

Developing molecular carriers for demethylase inhibitors has emerged as a promising strategy for targeted tumour cell therapy. GNPIPP12MA, a nanoparticle‐based delivery system, specifically targets leukaemic stem cells (LSCs), inducing apoptosis and CD8+ T cell infiltration. Its efficient uptake by AML cells and LSCs minimizes cytotoxicity to normal cells, enhancing therapeutic efficacy and reducing side effects.^214^ Similarly, loading IOX1, the inhibitor of ALKBH5, onto ferritin nanocage can strongly alleviate the damage of acute myocardial infarction, improve cardiac function and reduce infarct size.^215^

### 3.3 Inhibition of methylation recognition proteins activity

Among all “readers” so far, only IGF2BP1 has had inhibitors developed. Three IGF2BP1 inhibitors have been developed, including AVJ16, BTYNB and Cucurbitacin B (CuB). They have been shown to prevent the binding of IGF2BP1 and m6A to inhibit tumour progression. AVJ16 inhibits the migration of non‐small‐cell lung cancer cells.^216^ BTYNB strongly selectively inhibits the proliferation of IGF2BP1‐positive cells and suppresses the growth of many tumours.^217^, ^218^ CuB regulates the conformation of IGF2BP1 via Cys253 to reduce the stability of m6A mRNA, thereby promoting the migration of immune cells into the TME and blocking the expression of PD‐L1 which leads to the inhibition of hepatocellular carcinoma.^219^

### 3.4 Activation of methyltransferases

As discussed above, upregulating m6A levels by inhibiting demethylases has been postulated to be an effective strategy to delay disease progression. Conversely, activating methyltransferases offers an alternative strategy to elevate m6A levels. Piperidine and piperazine derivatives have emerged as potential drug candidates, with methyl piperazine‐2‐carboxylate demonstrating the highest efficacy and remarkable low cytotoxicity even at high concentrations.^220^

Currently, few studies have investigated methyltransferase activators. The role of activators in diseases remains to be further explored, and the combination of activators with demethylase inhibitors should be further investigated.

## 4 CONCLUSIONS AND FUTURE PERSPECTIVES

As a critical component in protein synthesis, even minor alterations in tRNA abundance or nucleotide modification levels can disrupt protein translation and impact disease progression. As previously noted, methylation modifications are ubiquitous in tRNA, and despite the increasing number of studies on these modifications, the molecular mechanisms and biological roles of tRNA methylation changes are not yet fully understood. Advancements in science and technology have paved the way for the development of sequencing techniques with improved sensitivity and accuracy, enabling the single‐molecule detection and quantification of tRNA modification levels. This progress is crucial for gaining a comprehensive understanding of the intricate dynamics of tRNA modifications. In the future, levels of tRNA modifications could potentially serve as innovative biomarkers for the early diagnosis of diseases, offering new avenues for early intervention and treatment strategies.

Interestingly, the level of tRNA methylation modification appears to be a double‐edged sword. While the majority of these methylation modification regulators, including “writers”, “erasers”, and “readers”, contribute to disease progression, some studies have suggested that these regulators can also have inhibitory effects on disease advancement. This dual effect may stem from the ability of changes in tRNA methylation levels to impact the translation efficiency of specific proteins and activate various downstream pathways. The network of post‐transcriptional modification is intricate, and potential interactions between different tRNA modifications to exert additional roles warrant further investigation. Understanding these complexities could shed light on novel mechanisms underlying disease processes and provide insights into potential therapeutic targets.

Current therapeutic strategies targeting methylation modification regulators mainly concentrate on the development of inhibitors for “writers” and “erasers”. However, most of these regulatory factors not only regulate tRNA methylation levels but also influence other RNA modifications. Consequently, these inhibitors cannot exclusively target tRNA methylation without affecting other RNAs. Lower selectivity may result in the emergence of new diseases and cytotoxic effects. Therefore, there is a crucial need for the development of highly selective, low cytotoxicity, and efficient inhibitors that specifically target tRNA methylation, representing a vital direction for future research. Changes in tRNA methylation levels influence tRF production. Identifying disease‐associated tRFs and developing drugs that directly target these molecules present promising avenues for investigation. While current therapeutic strategies for methylation modification regulators predominantly focus on “writers” and “erasers” inhibitors, it is important to note that these factors also influence other RNA modifications, underscoring the importance of developing targeted therapies with enhanced specificity and reduced off‐target effects.

Overall, RNA modifications in tRNA, specifically m1A, m3C, m5C, m1G, m7G, m5U, and Nm modifications, play important roles in various biological processes, including metabolic processing, stability, protein interactions, and mitochondrial activities. While traditional drug discovery efforts have primarily targeted inhibiting enzyme activity, the emergence of enzyme activators has opened new avenues for therapeutic intervention. Nevertheless, the current research is still limited and several key questions need to be addressed. Ongoing research in tRNA modifications is expected to enhance our understanding of disease mechanisms, improve disease diagnosis and enable better prognosis.

## AUTHOR CONTRIBUTIONS

Zhijing Wu and Ruixin Zhou contributed to writing original draft, designing the tables and figures. Baizhao Li and Mingyu Cao contributed to data acquisition and editing. Wenlong Wang and Xinying Li contributed to conceptualization, final manuscript's review and correspondence.

## FUNDING INFORMATION

This research is supported by the National Natural Science Foundation of China (No. 82270835), the Young and Middle‐aged Doctor Research Project of the Beijing Bethune Public Welfare Foundation (No. Z04JKM2022E036), the Natural Science Foundation of Hunan Province of China (No. 2024JJ6664), and China Postdoctoral Science Foundation (No. GZC20233156).

## CONFLICT OF INTEREST STATEMENT

The authors declare that there are no conflicts of interest.

## Data Availability

Data sharing not applicable to this article as no datasets were generated or analysed during the current study.
